# Real-world digital implementation of the Psychosis Polyrisk Score (PPS): A pilot feasibility study

**DOI:** 10.1016/j.schres.2020.04.015

**Published:** 2020-12

**Authors:** Dominic Oliver, Giulia Spada, Amir Englund, Edward Chesney, Joaquim Radua, Abraham Reichenberg, Rudolf Uher, Philip McGuire, Paolo Fusar-Poli

**Affiliations:** a Early Psychosis: Interventions and Clinical Detection (EPIC) Lab, Department of Psychosis Studies, Institute of Psychiatry, Psychology and Neuroscience, King's College London, London, United Kingdom; b OASIS Service, South London and the Maudsley NHS Foundation Trust, London, United Kingdom; c Institute of Psychiatry, Psychology and Neuroscience, King's College London, London, United Kingdom; d Imaging of Mood- and Anxiety-Related Disorders (IMARD), Institut d'Investigacions Biomèdiques August Pi i Sunyer (IDIBAPS), CIBERSAM, Barcelona, Spain; e Department of Clinical Neuroscience, Centre for Psychiatry Research, Karolinska Institutet, Stockholm, Sweden; f Department of Psychiatry, Icahn School of Medicine at Mount Sinai, New York, NY, United States; g Department of Environmental Medicine and Public Health, Icahn School of Medicine at Mount Sinai, New York, NY, United States; h Frieman Brain Institute, Icahn School of Medicine at Mount Sinai, New York, NY, United States; i Department of Psychiatry, Dalhousie University, Halifax, NS, Canada; j Department of Brain and Behavioral Sciences, University of Pavia, Pavia, Italy

**Keywords:** Clinical high risk, Risk, Prediction, Environment, Polygenic risk, Implementation

## Abstract

**Background:**

The Psychosis Polyrisk Score (PPS) is a potential biomarker integrating non-purely genetic risk/protective factors for psychosis that may improve identification of individuals at risk and prediction of their outcomes at the individual subject level. Biomarkers that are easy to administer are direly needed in early psychosis to facilitate clinical implementation. This study digitally implements the PPS and pilots its feasibility of use in the real world.

**Methods:**

The PPS was implemented digitally and prospectively piloted across individuals referred for a CHR-P assessment (n = 16) and healthy controls (n = 66). Distribution of PPS scores was further simulated in the general population.

**Results:**

98.8% of individuals referred for a CHR-P assessment and healthy controls completed the PPS assessment with only one drop-out. 96.3% of participants completed the assessment in under 15 min. Individuals referred for a CHR-P assessment had high PPS scores (mean = 6.2, SD = 7.23) than healthy controls (mean = −1.79, SD = 6.78, *p* < 0.001). In simulated general population data, scores were normally distributed ranging from −15 (lowest risk, RR = 0.03) to 39.5 (highest risk, RR = 8912.51).

**Discussion:**

The PPS is a promising biomarker which has been implemented digitally. The PPS can be easily administered to both healthy controls and individuals at potential risk for psychosis on a range of devices. It is feasible to use the PPS in real world settings to assess individuals with emerging mental disorders. The next phase of research should be to include the PPS in large-scale international cohort studies to evaluate its ability to refine the prognostication of outcomes.

## 1 Introduction

Primary indicated prevention in individuals at Clinical High Risk for Psychosis (CHR-P) (Fusar-Poli, 2017) has the potential to alter the course of psychotic disorders (Fusar-Poli et al., 2017b). Current assessment tools, like the Comprehensive Assessment for At-Risk Mental States (CAARMS) and the Structured Interview for Psychosis-risk Syndromes (SIPS) have very good prognostic accuracy (area under the curve at 3 years: 0.9) which is comparable to that of other tests used in clinical medicine. However, while these instruments are effective in ruling out psychosis risk, they are sub-optimal for predicting psychosis risk (Fusar-Poli et al., 2015; Oliver et al., 2018), leading to a 22% transition risk within three years (Fusar-Poli et al., 2020). Furthermore, these psychometric tools alone are limited to the ascertainment of symptoms and are unable to accurately predict the disease course at an individual level (Fusar-Poli et al., 2018a, Fusar-Poli et al., 2018b). There is therefore a clear need to improve prognostic accuracy of the CHR-P assessment by supplementing the clinical assessment with additional information from electrophysiology, neurocognition, blood, neuroimaging, genetics or environmental risk/protective biomarkers.

A number of studies have demonstrated that structural neuroimaging can predict both transition to psychosis (accuracy 82%, (Koutsouleris et al., 2009); accuracy 84.2%, (Koutsouleris et al., 2012b)) and global functioning (accuracy 76.9%, (Koutsouleris et al., 2018)) at the individual subject level. However, the implementation of these biomarkers in clinical routine has been limited by the cost of attaining sufficiently large datasets (Fusar-Poli et al., 2018b) and of associated logistical challenges.

Genetic biomarkers for psychosis risk have also been investigated: psychotic disorders such as schizophrenia are highly genetic conditions, with a first-degree heritability of 64% (95%CI: 62–68%) (Lichtenstein et al., 2009). Single genetic alterations are unlikely to be useful to predict clinical outcomes in psychosis: polygenic risk scores encompassing several genetic alterations have been developed using data from large genome-wide association studies (GWAS). However, polygenic risk scores have so far managed to only explain 18% of the variance between cases of established schizophrenia and healthy controls (Schizophrenia Working Group of the Psychiatric Genomics Consortium, 2014) and only 9% of the variance between cases of first-episode psychosis and healthy controls (Vassos et al., 2017). In CHR-P individuals, polygenic risk scores have been tested for stratified predictions (Perkins et al., 2020), but again the proportion of variance explained still remain modest (12.3%). Therefore, the prognostic ability of polygenic risk scores needs to be supplemented by additional information before it can be considered for real-world individualised risk prediction.

Environmental data may be the most appropriate modality to supplement polygenic risk scores, because the aetiology of psychotic disorders involves direct genetic and environmental effects as well as their interaction. Numerous factors can modulate an individual's risk of developing psychosis in the general population (Radua et al., 2018), or in those meeting CHR-P criteria compared to healthy controls (P Fusar-Poli et al., 2017) or in CHR-P individuals transitioning to psychosis compared to those who do not transition (Oliver et al., 2019b). Emulating the polygenic risk score approach, environmental risk/protective factors for psychosis have been combined into a single measure: the Psychosis Polyrisk Score (PPS) (Oliver et al., 2019a).

The conceptual and methodological underpinnings for developing the PPS have been detailed previously in a conceptual review (Oliver et al., 2019a). The PPS incorporates environmental risk factors for psychosis (sociodemographic, social, parental, perinatal, later risk/protective factors and antecedents) which have been systematically appraised in an umbrella review and stratified according to their level of evidence for association with psychotic disorders (Radua et al., 2018). Demographic, parental, social, and perinatal risk factors are generally thought to exert their role during the early developmental phases (Davies et al., 2020), while later risk/protective factors and antecedents can modulate psychosis risk from late childhood up to shortly prior psychosis onset. Later risk factors indicate a passive exposure to socio-environmental factors, whereas antecedents involve active risk-modifying processes involved in psychosis onset and premorbid deviations in functioning (Dean and Murray, 2005; Matheson et al., 2011). However, these categories are only descriptive and may overlap.

The current study extends this line of research by digitally implementing the PPS and by conducting the first pilot feasibility study for its use in the real world. We also compared the distribution of PPS scores across three different groups: individuals referred for a CHR-P assessment, healthy controls and a simulated dataset representing the general population. We hypothesised that individuals referred for a CHR-P assessment would have the highest PPS scores and healthy controls the lowest. The findings of this study will inform subsequent research in this area, in particular the incorporation of the PPS in large scale prospective CHR-P cohort studies that are about to start.

## 2 Materials and methods

### 2.1 Participants

The study included two participant groups: individuals referred for a CHR-P assessment and healthy controls.

#### 2.1.1 Individuals referred for a CHR-P assessment

These individuals were sampled from those referred to Outreach and Support in South London (OASIS, (Fusar-Poli et al., 2013; Fusar-Poli et al., 2019a)) a CHR-P service, part of the South London and Maudsley NHS Foundation Trust (SLaM) from secondary mental health services on suspicion of psychosis risk. They were all under SLaM care for a non-psychotic mental disorder. These individuals were included if they i) had been referred for a CHR-P assessment by SLaM mental health services; ii) were older than 14 years of age, with no upper age limit (to capture pathways to care within SLaM early intervention services in a lifespan-inclusive approach); iii) were willing and able to provide written informed consent; iv) had sufficient understanding of the English language; v) had no diagnoses of organic psychiatric disorders, substance-induced psychotic disorders or psychotic disorders (with the exception of Acute and Transient Psychotic Disorders; ATPD; eMethods 1), as ascertained by SLaM clinicians. In addition to the PPS, standard psychometric tools including the CAARMS and Global Assessment of Functioning (GAF) were administered.

#### 2.1.2 Healthy controls

Healthy controls were recruited from a separate study completed at King's College London via university circular emails. Participants met inclusion criteria if they i) were aged over 14, with no upper age limit; ii) were willing and able to provide written informed consent; iii) had sufficient understanding of the English language, iv) did not have a past or present mental disorder (any type) or psychotic-like symptoms or physical illness, as detected through the mini-Structured Clinical Interview for DSM-IV (mini-SCID), Adolescent Psychotic-like Symptom Screener (APSS) (Kelleher et al., 2011) or physical examination; v) had never been treated with antipsychotic or antidepressant medication at any dosage; vi) did not have a first degree relative affected by a psychotic illness (assessed by the mini-SCID).

### 2.2 Operationalisation of the PPS

#### 2.2.1 Selection of risk factors

To develop the PPS, we initially considered all 17 risk factors that were meeting the highest hierarchy of evidence (i.e. class I–III) for association with psychotic disorders, as detailed in the umbrella review (Radua et al., 2018). We then excluded those factors (n = 6) that could not easily be measured at scale due to cost (Toxoplasma Gondii IgG), extended assessment time (premorbid IQ) or limited reliability of self-report (olfactory identification ability, minor physical anomalies). We also excluded the CHR-P state to investigate the PPS independently from this construct. This left 12 remaining class I-III risk factors (eMethods 2). In addition to these risk factors, we additionally included some class IV risk factors that could be recorded at low cost, high reliability and limited assessment time (n = 11, eMethods 2). A final number of 22 factors were eventually operationalised in the PPS (eMethods 2).

To ensure accurate scoring that was reflective of the risk factor as reported in the umbrella review (Radua et al., 2018), the same tools were used where possible to assess the presence/absence of the risk factor as the studies from which the risk was reported. The tools used to ascertain the presence/absence of each risk factor as well as details of operationalisations and the cut-offs for defining each respective Odds Ratio (OR) of included factors can be seen in eTable 1.

#### 2.2.2 Integration of different risk factors in the PPS

Similar to the polygenic risk score, the PPS is a weighted sum of exposure to risk and protective factors, using the ORs associated with each factor (Radua et al., 2018) (see eMethods 3).

Simple adaptations were added to the model to account for interdependencies in exposures, such as the multiple risk/protective factors associated with immigration status (see Table 1). For example, individuals cannot be exposed to certain immigration-based risk/protective factors in conjunction with each other. Immigrants cannot be both first-generation and second-generation, and North African immigrants have to be either first- or second-generation immigrants. We combined these factors following this logic and assuming that the proportion and extra risk of North African immigrants is similar in first- and second-generation immigrants (Eurostat (European Commission), 2015). Factors related to ethnicity have similar logical dependencies between them, e.g. black Caribbean is a non-white ethnicity, and individuals cannot be from a low ethnic density area, from a medium density area and from a high ethnic density area at the same time. We combined these factors again following this logic and assumed that the proportion and extra risk of black Caribbean individuals between non-white ethnicity individuals is similar in low, medium and high ethnic density areas. Following these combinations, the final scoring of these factors can be seen in Table 1). Limitations of the PPS were previously presented (Oliver et al., 2019a).

**Table 1:** Scoring system for the Psychosis Polyrisk Score (PPS).

Risk/protective factor	Exposure/conditional		PPS
Childhood trauma	Yes		4
	No		−0.5
Ethnicity	White		−2
	Black Caribbean	In low ethnic density area	6
		In medium ethnic density area	5.5
		In high ethnic density area	3.5
	Other	In low ethnic density area	3.5
		In medium ethnic density area	3
		In high ethnic density area	1
Immigration	Not immigrant		−0.5
	1st generation immigrant	From North Africa	3
		From other regions	2
	2nd generation immigrant	From North Africa	2.5
		From other regions	1.5
Non-right-handedness	Yes		2
	No		0
Pollution	Yes		2
	No		−5.5
Urbanicity	Yes		1
	No		−2.5
Winter or spring birth in northern hemisphere	Yes		0
	No		0
Paternal age	<35		−0.5
	>35		0.5
	>45		3.5
Low paternal socioeconomic status	Yes		1
	No		0
Parental severe mental illness	Yes		5.5
	No		−2
Adult life events	Yes		5.5
	No		−2
Daily smoker	Yes		3
	No		−0.5
Heavy cannabis use	Yes		7
	No		0
Hearing problems in past 12 months	Yes		2
	No		0
Trait anhedonia	Yes		6.5
	No		0
Male & 25–35 years old	Yes		2
	No		0

### 2.3 Real-world digital implementation of the PPS assessment

Feasibility is one of the core deliverables of the PPS, as risk estimation systems are of little value without real-world usability (McGorrian et al., 2012). To enhance this aspect, the PPS was designed as a self-report assessment, minimising researcher/clinician burden. Moreover, the PPS was implemented online to facilitate its administration on tablets (as it was in this study), computers or phones. This provides options for users and, alongside a progress saving feature, increases likelihood of completed assessments as individuals can complete them on any device across multiple sessions.

To characterise the administration properties of the PPS, at the end of the assessment, participants were asked how long the assessment took to complete (less than fifteen minutes, 15–30 min, 30–45 min or >45 min) and how distressing they found the process of completing the questionnaire (not distressing at all, mildly distressing, very distressing or extremely distressing).

### 2.4 Statistical analysis

Baseline clinical and sociodemographic characteristics of the participants (individuals referred for a CHR-P assessment and healthy controls) were described by means and standard deviations for continuous variables, and absolute and relative frequencies for categorical variables. Differences between continuous sociodemographic/clinical variables in the two participant groups were assessed using independent sample *t*-tests; differences between categorical sociodemographic/clinical variables were assessed using Fisher's exact test.

To further estimate the usability of the PPS in the wider scenario, we built a simulated dataset to investigate the range and distributions of PPS scores in the general population. This additionally allows for greater comparability with the pilot PPS presented in our previous paper (Oliver et al., 2019a). In a first step, we ran 10,000,000 permutations for each PPS risk factor using general population prevalence data that best represented the sample from which the ORs were generated from (eTable 2). In a second step, a PPS score was generated for each permutation, enabling investigation of the range and distribution of PPS scores in the general population. Normality of distribution was investigated using an adjusted Jarque-Bera test.

We then tested the differences in PPS scores between the three groups (individuals referred for a CHR-P assessment, healthy controls and simulated general population) using one-way analysis of variance (ANOVA) and post-hoc Tukey Honest Significant Differences test. We also performed correlation tests in individuals referred for a CHR-P assessment using Pearson's R to investigate potential correlations between PPS and CAARMS total scores. All analyses were conducted in R version 3.3.2. (R Core Team, 2014) and significance was set to *p* < 0.05.

## 3 Results

### 3.1 Characteristics of study participants

Sixteen individuals referred for a CHR-P assessment were recruited onto the study. One of them was unable to complete the assessment due to fatigue, leaving a final sample of fifteen individuals. Three individuals (20.0%) were presenting with a diagnosis of ATPD and the remaining 12 (80.0%) with bipolar mood disorders. Two individuals (13.3%) met CHR-P criteria with a further two (13.3%) meeting CAARMS criteria for psychosis. Mean age was 36.7 (SD = 12.7) and 26.7% were male (Table 2).

**Table 2:** Sociodemographics of study participants: individuals referred for a CHR-P assessment and healthy controls.

	Individuals referred for a CHR-P assessment (n = 15) Mean (SD)	Healthy controls (n = 66) Mean (SD)	*p*
Age, y	36.7 (12.7)	25.9 (4.9)	0.006
Ethnicity No. (%White)	6 (40.0%)	52 (78.8%)	0.008
Gender No. (%Male)	4 (26.7%)	30 (45.5%)	0.25
Index diagnosis No. (%)			
Acute and transient psychotic disorders	3 (20.0%)	N/A	N/A
Bipolar mood disorders	12 (80.0%)	N/A	N/A
CAARMS P1-P4	11.29 (5.59)	N/A	N/A
No. meeting CHR-P criteria (%)	2 (13.3%)	N/A	N/A
No. meeting psychosis criteria (%)	2 (13.3%)	N/A	N/A
GAF	71 (11.71)	N/A	N/A

Abbreviations: CAARMS Comprehensive Assessment for At-Risk Mental States P1-P4 domains; GAF Global Assessment of Functioning.

Sixty-six healthy controls were recruited onto the study. Mean age was 25.9 (SD = 4.9) and 45.5% were male (Table 2).

### 3.2 Digital implementation and feasibility of administration

The PPS has been integrated online (https://www.youngspace.org) as a digital assessment tool that can be presented on multiple devices. 15/16 individuals referred for a CHR-P assessment (93.7%) were able to complete the digital self-report PPS with a single participant unable to complete due to fatigue. All 66 healthy controls completed the PPS assessment. Overall 81/82 participants (98.8%) were able to complete the assessment. 78 (96.3%) participants completed the assessment in under 15 min. No participants found the content of the assessment distressing.

### 3.3 PPS scores in individuals referred for a CHR-P assessment

PPS scores in this group ranged from −3 (RR = 0.5) to 18 (RR = 63.10). Mean PPS score was 6.2 (SD = 7.23) The median PPS score in this group was 9 (RR = 7.94). The PPS distributions in the quartile ranges were Q1 (−3 to −1.5): 26.7%; Q2 (−1 to 9): 33.3%; Q3 (9.5 to 11.5): 20%; Q4 (12 to 18): 20%. 60.0% of participants in this group had a PPS > 5 (RR > 3) with 6.7% having a PPS > 15 (RR > 30). Correlations between PPS and CAARMS total scores were non-significant (*p* = 0.26; eFig. 1).

### 3.4 PPS scores in healthy controls

PPS scores in this group ranged from −14 (RR = 0.04) to 14.5 (RR = 28.18). Mean PPS score was −1.79 (SD = 6.78). The median PPS score in this group was −1.75 (RR = 0.67). The PPS distributions in the quartile ranges were Q1 (−14 to −7): 27.3%; Q2 (−6.5 to −1.75): 22.7%; Q3 (−1.5 to 1.875): 24.2%; Q4 (2 to 14.5): 25.8%.

19.7% of participants in this group had a PPS > 5 (RR > 3) with 0% having a PPS > 15 (RR > 30).

### 3.5 PPS scores in the simulated general population

**Fig. 1:**
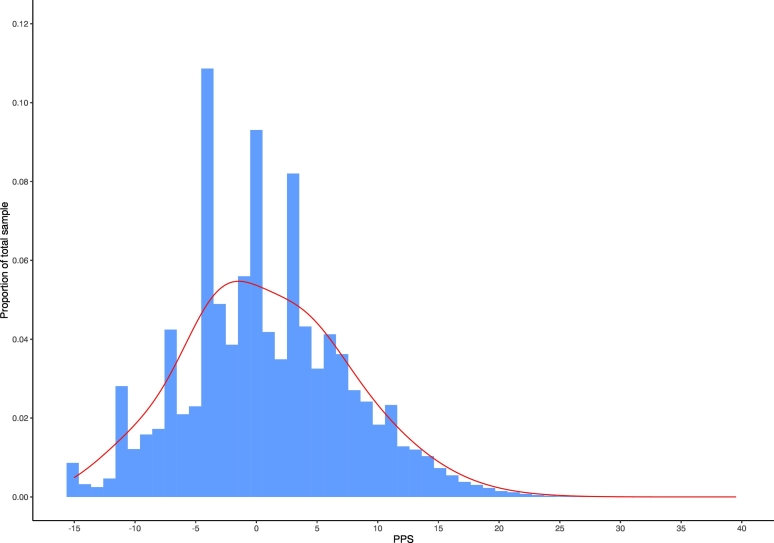
Distribution of PPS scores in the simulated general population. Blue bars indicate the proportion of individuals receiving each PPS score based on the prevalence of risk factors and 10,000,000 permutations. Red line indicates the density curve to highlight normality. (For interpretation of the references to colour in this figure legend, the reader is referred to the web version of this article.)

PPS scores in this simulated group ranged from −15 (lowest risk; RR = 0.03) to 39.5 (highest risk; RR = 8912.51) (Fig. 1). These scores were normally distributed (AJB = 104,030, *p* < 0.001). Mean PPS score in this simulated group was 0.817 (SD = 6.87). The median PPS score was 0 (RR = 1). The PPS distributions in the quartile ranges were Q1 (−15 to −4): 29.0%; Q2 (−3.5 to 0): 21.0%; Q3 (0.5 to 5.5): 26.5%; Q4 (6 to 39.5): 23.5%. 26.7% of individuals in this simulated group had a PPS > 5 (RR > 3) with 2.7% of individuals having a PPS > 15 (RR > 30).

### 3.6 Comparison of PPS score distribution between individuals referred for a CHR-P assessment, healthy controls and the general population

**Fig. 2:**
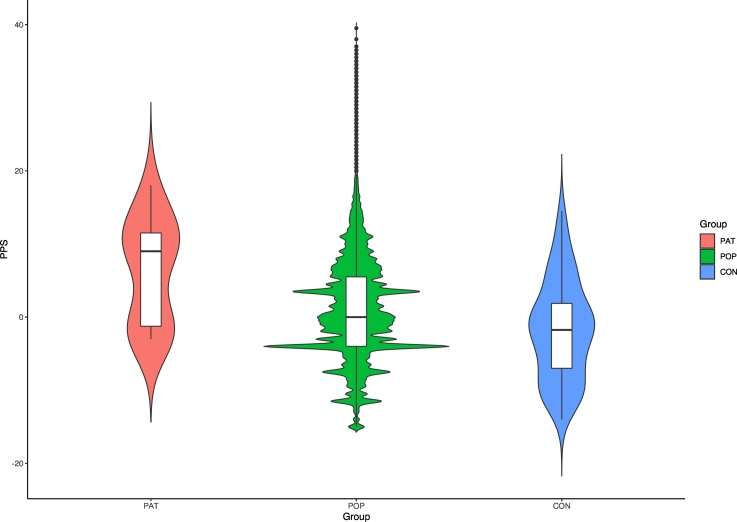
Violin plot comparing PPS scores distribution in individuals referred for a CHR-P assessment, controls and the (simulated) general population. PAT, patients referred for a CHR-P assessment; POP, simulated general population; CON, healthy controls.

There was a main effect of group on PPS scores (F_2,10__,__000__,__000_ = 9.357, *p* = 0.001). Individuals referred for a CHR-P assessment had higher PPS scores (mean = 6.2, SD = 7.23) than healthy controls (mean = −1.79, SD = 6.78) (p < 0.001) and the simulated general population dataset (mean = 0.817, SD = 6.87; *p* = 0.007). PPS scores in the simulated general population were higher than healthy controls (*p* = 0.006) (Fig. 2).

## 4 Discussion

This pilot study digitally implemented the PPS and illustrates its real-world feasibility of use in different scenarios. Additionally, it supports its potential as a biomarker to complement current assessment tools and improve both identification of individuals at risk of psychosis and prediction of their clinical outcomes.

The results of this pilot feasibility study reinforce the theoretical strengths of the PPS biomarker on a pragmatic implementation level. The digital implementation of the PPS highlights its future real-world usability, particularly through its ease of administration. However, further large-scale studies are needed to confirm whether the PPS can reliably identify individuals at risk for psychosis or improve the prediction of their outcomes. Digital assessments reduce costs and increase data completeness (Ebert et al., 2018), take less time than paper assessments (Khraishi et al., 2012) and are usually preferred by participants (Salaffi et al., 2013). Additionally, simulated data in the general population displayed a broad range of scores, suggesting a high level of potential variance in future studies, which is imperative for individualised prediction. There is currently high heterogeneity within the CHR-P construct with some CHR-P individuals at extremely high risk of transition to psychosis (e.g. Brief Limited Intermittent Psychotic Symptoms [BLIPS] (Fusar-Poli et al., 2016; Fusar-Poli et al., 2017a)) and others who barely differ from the general population (e.g. Genetic Risk and Deterioration [GRD] (Fusar-Poli et al., 2016)). While these subgroups can be used to stratify people to some degree, this approach is sub-optimal as high heterogeneity remains within the largest subgroup (85% of CHR-P individuals; (Attenuated Psychotic Symptoms [APS], (Fusar-Poli et al., 2016)). The high variance in PPS scores in this simulated dataset and independence of PPS and CAARMS total scores support the potential of the PPS to refine estimates of psychosis risk, however this needs to be confirmed by future studies. This allows for transdiagnostic detection of individuals at risk for psychosis beyond the CHR-P approach (Fusar-Poli, 2019; Fusar-Poli et al., 2019c). Further to this, the PPS was not age-restricted, instead adopting a lifespan-inclusive approach, allowing for an extended CHR-P phenotype unrestricted by age. The PPS concept was built on the assumption that as individuals accumulate environmental risk factors for psychosis through the various stages of help-seeking and disorder progression (Fusar-Poli et al., 2017; Radua et al., 2018), which is supported by the higher scores being seen in individuals referred for a CHR-P assessment than in healthy controls. The PPS is also robust, as risk factors were selected systematically a priori. As the weighting of the PPS risk factors was determined a priori by umbrella review, the gold standard of evidence (Fusar-Poli et al., 2018b), the PPS is therefore based on data from a combination of different samples in different settings, reducing risk of overfitting issues and improving generalisability. The PPS is also optimisable: the knowledge base is dynamic and changing, which allows for weights of existing factors to be adjusted as new evidence becomes available or for new predictors to be added to the model, particularly more dynamic factors, e.g. symptom severity at multiple time points (Studerus et al., 2020), digital phenotyping measures (Reilly et al., 2019) or automated speech analysis (Corcoran et al., 2018), which allow for repeated use and dynamic mapping. Similarly, if those factors that were previously considered to be impractical to measure exposure to (e.g. Toxoplasma Gondii) become more feasible, they could also be integrated.

The main application of the PPS biomarker may be to improve the detection of individuals at risk for psychosis (Fusar-Poli et al., 2019d). Despite considerable effort to improve the detection of CHR-P individuals, only 5–12% of first episode of psychosis cases are detected prior to psychosis onset (Fusar-Poli et al., 2017c; McGorry et al., 2018). Furthermore, up to one third of first episode cases do not experience a prodrome (Häfner et al., 2003; Schultze-Lutter et al., 2015; Shah et al., 2017). The PPS could potentially then provide an adjunct to current symptom-based risk ascertainment strategies to identify individuals at risk who do not experience CHR-P features such as attenuated or intermittent psychotic symptoms before their first episode. The PPS could also supplement digital detection strategies such as the Youth-Mental Risk and Resilience study (YouR-Study) (McDonald et al., 2019). The YouR-Study integrated an online screening tool encompassing the Prodromal Questionnaire (PQ-16) (Ising et al., 2012) and a nine-item scale of perceptual and cognitive aberrations. Out of 2279 participants completing the assessment, 78% met a risk threshold were invited to attend a clinical assessment, 356 interviews were completed, 28% of these individuals met CHR-P criteria and 2% met criteria for first episode psychosis at this assessment (McDonald et al., 2019). This study provided the first evidence of feasibility of digital detection tools improving identification of psychosis in the general population but performance in terms of sensitivity/specificity (81%/57%) of the screener could be improved upon. The YouR-Study approach could be combined with the PPS and the additional information could produce a more accurate screening tool and improve detection of CHR-P individuals. Integration of the PPS within YouR-Study is being planned as part of an ongoing project.

The above targets can be fully accomplished within a stepped assessment strategy which stratifies individuals' risk of developing psychosis. Leveraging different assessment types in sequence could be a pragmatic method that greatly benefits risk stratification. For example, while Electronic Health Records are not data rich enough to allow for automated screening of PPS variables on a large scale—a goal achieved using risk prediction models that incorporate predictors collected routinely in clinical care (Fusar-Poli et al., 2017c; Fusar-Poli et al., 2019b, Fusar-Poli et al., 2019e)—the use of natural language processing methods may fill this gap. The PPS biomarker, if integrated into Electronic Health Records or website approaches (such as YouR-Study) could then become the first entry point in a stepped risk stratification framework. More labour- and time-intensive biomarkers, such as those that rely on cognitive assessments (Cannon et al., 2016), clinical assessments (Cannon et al., 2016; Leighton et al., 2019a; Leighton et al., 2019b) or neuroimaging (Koutsouleris et al., 2009; Koutsouleris et al., 2012a, Koutsouleris et al., 2015; Koutsouleris et al., 2018) could be reserved to those individuals initially detected through PPS screening (Fusar-Poli et al., 2019b). However, more work needs to be done to assess the prognostic ability of the PPS. A previous study has demonstrated the potential predictive gain in sequential testing in this manner, with clinical and electrophysiological biomarkers following an initial CHR-P assessment, followed by subsequent structural MRI and blood biomarkers, with an individual only progressing to the next risk testing stage if the previous test was positive (Schmidt et al., 2017).

The main limitation of this study is the small sample and lack of external validation of the PPS. Since we did not report data on transition to psychotic disorders, we were unable to produce measures of prognostic accuracy; the small sample size would have prevented meaningful analyses. The study is similarly underpowered to compare referrals who met CHR-P criteria and those who did not or to test correlations with psychopathology or other clinically relevant variables. Future efforts are clearly needed to further dissect the heterogeneity of CHR-P outcomes. This would clearly require establishing large scale datasets with enough power and follow-up time to assess the validity of the PPS. A potential research framework that may validate the PPS biomarker is the proposed 26-site ProNET cohort study. Other large-scale international collaborations that have recently been completed include the HARMONY project, incorporating NAPLS (https://campuspress.yale.edu/napls/, (Addington et al., 2012)), PRONIA (https://www.pronia.eu/) and PSYSCAN (http://psyscan.eu, (Tognin et al., 2020)). Another crucial limitation is that it is also unlikely that the risk factors used in the PPS are independent as, assumed. Further work emerging from the above international consortia will clarify how the PPS risk factors interact.

## 5 Conclusions

The digital implementation of the PPS as a self-assessed biomarker facilitates its real-world use in diverse scenarios. The PPS biomarker holds theoretical potential for improving the detection of individuals at risk and prediction of their outcomes, in particular if used within a stepped risk assessment framework. Future large-scale international consortia are needed to validate the PPS prospectively.

## Contributions

D.O., G.S., A.E. and E.C. collected the data. D.O. performed all data analysis and drafted the first version of the manuscript. J.R. advised on data analysis. D.O., A.R., R.U., P.M. and P.F.-P. advised on study design and interpretation of results. P.F.-P. designed and supervised the study. All authors read and edited the manuscript.

## Declaration of competing interest

P.F.-P. has received advisory consultancy fees from Lundbeck outside of this work. The authors have declared that there are no conflicts of interest in relation to the subject of this study.

## Appendix A Supplementary data

Supplementary material Image 1
