# Supplementary material for: Real-world digital implementation of the Psychosis Polyrisk Score (PPS): A pilot feasibility study
Source: Schizophr Res. 2020 Dec;226:176–83. doi: 10.1016/j.schres.2020.04.015 (PMC7774585; doi:10.1016/j.schres.2020.04.015)
Supplement: Supplementary file 1 — Supplementary material [file mmc1.docx]

**Supplementary Online Content**

Oliver D, Spada G, Englund A et al. Psychosis Polyrisk Score (PPS): a pilot study to improve detection of individuals at-risk and prediction of clinical outcomes

**eMethods 1** Rationale for inclusion of acute and transient psychotic disorders

**eMethods 2** List of included risk/protective factors from umbrella review

**eMethods 3** Details of PPS construction

**eTable 1** List of included factors, along with their definitions and the tentative cut-offs for defining each respective Risk Ratio

**eTable 2** Prevalence of factors used in PPS in the general population

**eFigure 1** Correlation of PPS scores and CAARMS Total

**eReferences**

**eMethods 1** Rationale for inclusion of acute and transient psychotic disorders

The acute and transient psychotic disorders (ATPD) group was not considered a psychotic disorder because it is diagnostically (Fusar-Poli et al., 2017a) and prognostically (Fusar-Poli et al., 2016a) similar to the Brief Limited Intermittent Psychotic Symptoms (BLIPS) subgroup of the CHR-P construct (for details on these competing operationalisations, see eTable 1 in (Fusar-Poli et al., 2016a)).

**eMethods 2** List of included risk/protective factors from umbrella review

*Class I*

1. Black‐Caribbean ethnicity in England

*Class II*

1. Ethnic minority in low ethnic density area
2. Second generation immigrants
3. Trait anhedonia

*Class III*

1. Childhood trauma
2. Ethnic minority in high ethnic density area
3. First generation immigrants
4. Non‐right handedness
5. North African immigrants in Europe
6. Urbanicity
7. Winter/spring season of birth in Northern hemisphere

*Class IV*

1. Adult life events
2. Asian ethnicity in England
3. Black African ethnicity in England
4. Disadvantaged vs. advantaged groups
5. Heavy cannabis use
6. Low paternal socio‐economic status
7. Mixed ethnicity in England
8. Other White ethnicity in England
9. Parental severe mental illness
10. Tobacco use
11. Traffic

**eMethods 3** Details of PPS construction

To construct the PPS, we first estimated a raw score for each factor as the 10-base logarithm of its OR. For example, the OR of psychosis in individuals living in urban settings is 2.2, and thus the raw score of the urbanicity factor was log10(2.2) = 0.34. We then subtracted the population average of this raw score, so that individuals at risk would have positive scores and the remaining individuals would have negative scores, with an average of zero. For example, given that ~73.6% individuals live in urban settings worldwide (and thus 26.4% in rural settings with a raw score of 0), the population average of the urbanicity factor should be (73.6% × 0.34) + (26.4% × 0) = 0.25. We subtracted this average from the raw scores, i.e., the subtracted score was 0.34–0.25 = 0.09 for individuals in urban settings and 0–0.25 = −0.25 for individuals in rural settings. Further information about prevalence data used can be seen in eTable 2. Finally, for the ease of use we multiplied the subtracted scores by 10 and rounded them to the nearest half integer. In the example, the final scores were 0.09 × 10 ≈ 1 for individuals in urban settings and −0.25 × 10 = −2.5 for individuals in rural settings.

| **eTable 1** List of included factors, along with their definitions and the tentative cut-offs for defining each respective Risk Ratio | | | | |
| --- | --- | --- | --- | --- |
| **Risk factor assessed (Classification of evidence; Meta-analytical reference)** | **Assessment tool; Reference** | **Scoring (Classification of evidence; meta-analytical reference)** | **Cutoff** | **PPS scoring** |
| Age/Gender^3,4^ (IV) |  |  | Aged 25-35 & Male | Yes: 2  No: 0 |
| Non-right-handedness^5^ (III) |  |  |  | Yes: 2  No: 0 |
| Pollution^6^ (IV) |  | UK census data | 90^th^ percentile of per capita emissions at local authority level | Yes: 2  No: -5.5 |
| Urbanicity^7^ (III) |  | UK census data | Over 50% population in urban areas | Yes: 1  No: -2.5 |
| Ethnic density |  | UK census data | Low: Lowest 33^rd^ percentile  Medium: Middle 33^rd^ percentile  High: Highest 33^rd^ percentile | N/A – combine with ethnicity below |
| Black Caribbean Ethnicity^8^ (I) |  | In low ethnic density area^9^ (II) |  | 6 |
|  |  | In medium ethnic density area |  | 5.5 |
|  |  | In high ethnic density area^9^ (III) |  | 3.5 |
| Other Ethnicity^4^ (IV) |  | In low ethnic density area^9^ (II) |  | 3.5 |
|  |  | In medium ethnic density area |  | 3 |
|  |  | In high ethnic density area^9^ (III) |  | 1 |
| White |  |  |  | -2 |
| Not immigrant |  |  |  | -0.5 |
| 1st generation immigrant^10^ (III) |  | From North Africa^11^ (III) |  | 3 |
|  |  | From other regions |  | 2 |
| 2nd generation immigrant^10^ (II) |  | From North Africa^11^ (III) |  | 2.5 |
|  |  | From other regions |  | 1.5 |
| Paternal age <35 |  |  |  | -0.5 |
| Paternal age > 35^12^ (IV) |  |  |  | 0.5 |
| Paternal age > 45^12^ (IV) |  |  |  | 3.5 |
| Paternal SES^13^ (IV) | SES-Child^14^ | - *Higher executive, proprietor of large businesses, major professional = 0* - *Administrators, lesser professionals or proprietor of medium-sized business = 0.125* - *Smaller business owner, farm owner, manager or minor professional = 0.25* - *Technician, semi-professional or small business owner (business valued at £50,000-70,000) = 0.325* - *Clerical and sales worker, small farm or business owner (business valued at £25,000-50,000) = 0.5* - *Smaller business owner (<£25,000), skilled manual labourer, craftsman or tenant farmer = 0.625* - *Machine operator or semi-skilled worker = 0.75* - *Unskilled worker = 0.875* - *Farm labourer, menial service worker, student, dependent on welfare or no regular occupation = 1* - *Not applicable or unknown = 1* | ≥0.75 | Yes: 1  No: 0 |
| Parental Severe Mental Illness^15^ (IV) | Family Interview for Genetic Studies [FIGS]^16^ |  | ≥1 | Yes: 5.5  No: -2 |
| Adult Life Events^17^ (IV) | Life Threatening Events Questionnaire [LTE-Q]^18^ |  | >0 | Yes: 5.5  No: -2 |
| Tobacco use^19^ (IV) |  |  | Daily smoker | Yes: 3  No: -0.5 |
| Heavy cannabis use^20^ (IV) |  |  | More than once a week or every day | Yes: 7  No: 0 |
| Childhood Trauma^21^ (III) | Childhood Trauma Questionnaire Short Form [CTQ-SF]^22^ | *Never true = 1, rarely true = 2, sometimes true = 3, often true = 4, Very often true =5. None = 5-40, Low = 41-55; Moderate = 56-72; Severe = 73+* | Moderate and above | Yes: 4  No: -0.5 |
| Trait Anhedonia^23^ (II) | Temporal Experience of Pleasure Scale [TEPS]^24^ | Very true for me = 1, Often true for me = 2, Sometimes true for me = 3, Sometimes false for me = 4, Often false for me = 5, Very false for me = 6; items 51 is reverse scored | >35 | Yes: 6.5  No: 0 |

| **eTable 2** Prevalence of factors used in PPS in the general population | | |
| --- | --- | --- |
| **Factor** |  | **Prevalence** |
| Black Caribbean ethnicity in England^25^ |  | 3.462% |
| Ethnic minority in low ethnic density area^25^ |  | 20.85% |
| 2nd generation immigrant^26^ |  | 9.2% |
| Childhood trauma^27^ |  | 11.7560497% |
| North African immigrants in Europe^25^ |  | 0.0023% |
| Urbanicity^28^ |  | 73.6% |
| Ethnic minority in high ethnic density area^25^ |  | 67.99% |
| 1st generation immigrant^26^ |  | 16.8% |
| Non-right handedness^29^ |  | 5.9% |
| Seasonality of birth in Northern hemisphere |  | 50% |
| Paternal age^12^ | <35 | 77% |
|  | >35 | 20% |
|  | >45 | 3% |
| Paternal socioeconomic status^30^ | Low | 10% |
|  | Not low | 90% |
| Parental severe mental illness^15^ | Yes | 26% |
|  | No | 74% |
| Adult life events^17^ | Yes | 25% |
|  | No | 75% |
| Daily smoker^31^ | Yes | 19% |
|  | No | 81% |
| Heavy cannabis use^32^ | Yes | 3.50% |
|  | No | 96.50% |
| Hearing problems in past 12 months^33^ | Yes | 11% |
|  | No | 89% |
| Trait anhedonia^34^ | Yes | 1% |
|  | No | 99% |
| Male & 25-35yo^35^ | Yes | 7% |
|  | No | 93% |
| Pollution^36^ | Yes | 74% |
|  | No | 26% |

**eFigure 1** Correlation of PPS scores and CAARMS Total **
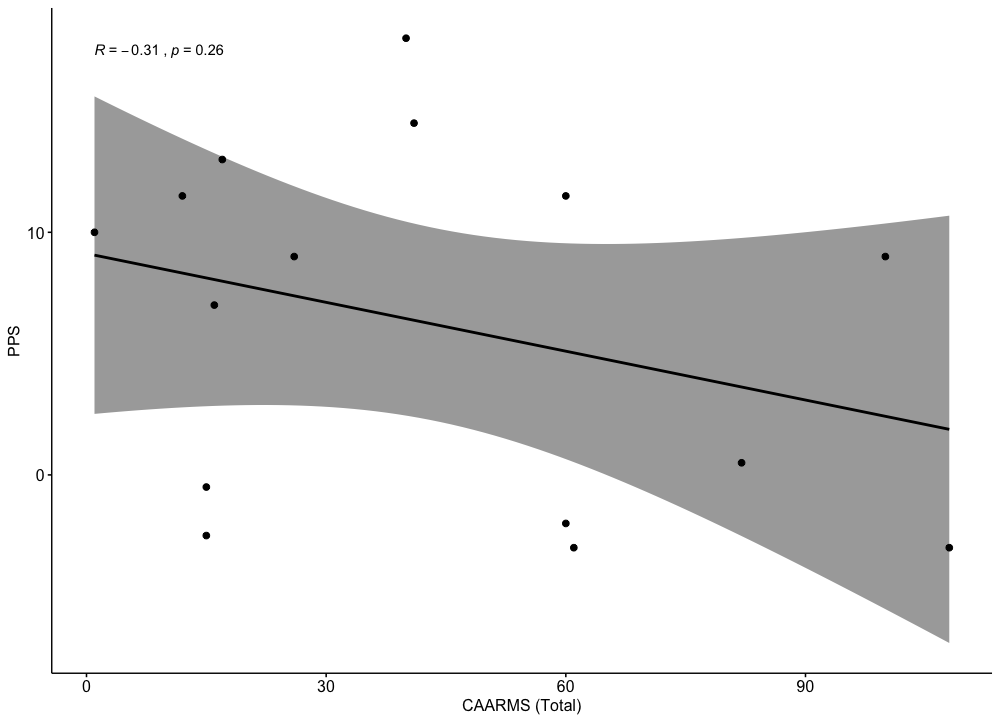
**

**eReferences**

1 Fusar-Poli P, Cappucciati M, De Micheli A, *et al.* Diagnostic and prognostic significance of brief limited intermittent psychotic symptoms (BLIPS) in individuals at ultra high risk. *Schizophr Bull* 2017; **43**: 48–56.

2 Fusar-Poli P, Cappucciati M, Bonoldi I, *et al.* Prognosis of Brief Psychotic Episodes: A Meta-analysis. *JAMA Psychiatry* 2016; **73**: 211–20.

3 Jackson D, Kirkbride J, Croudace T, *et al.* Meta-analytic approaches to determine gender differences in the age-incidence characteristics of schizophrenia and related psychoses. *Int J Methods Psychiatr Res* 2013; **22**: 36–45.

4 Kirkbride JB, Errazuriz A, Croudace TJ, *et al.* Incidence of schizophrenia and other psychoses in England, 1950-2009: a systematic review and meta-analyses. *PLoS One* 2012; **7**: e31660.

5 Hirnstein M, Hugdahl K. Excess of non-right-handedness in schizophrenia: meta-analysis of gender effects and potential biases in handedness assessment. *Br J Psychiatry* 2014; **205**: 260–7.

6 Attademo L, Bernardini F, Garinella R, Compton MT. Environmental pollution and risk of psychotic disorders: A review of the science to date. *Schizophr Res* 2017; **181**: 55–9.

7 Vassos E, Pedersen CB, Murray RM, Collier DA, Lewis CM. Meta-analysis of the association of urbanicity with schizophrenia. *Schizophr Bull* 2012; **38**: 1118–23.

8 Tortelli A, Errazuriz A, Croudace T, *et al.* Schizophrenia and other psychotic disorders in Caribbean-born migrants and their descendants in England: systematic review and meta-analysis of incidence rates, 1950-2013. *Soc Psychiatry Psychiatr Epidemiol* 2015; **50**: 1039–55.

9 Bosqui TJ, Hoy K, Shannon C. A systematic review and meta-analysis of the ethnic density effect in psychotic disorders. *Soc Psychiatry Psychiatr Epidemiol* 2014; **49**: 519–29.

10 Bourque F, van der Ven E, Fusar-Poli P, Malla A. Immigration, social environment and onset of psychotic disorders. *Curr Pharm Des* 2012; **18**: 518–26.

11 van der Ven E, Veling W, Tortelli A, *et al.* Evidence of an excessive gender gap in the risk of psychotic disorder among North African immigrants in Europe: a systematic review and meta-analysis. *Soc Psychiatry Psychiatr Epidemiol* 2016; **51**: 1603–13.

12 Torrey EF, Buka S, Cannon TD, *et al.* Paternal age as a risk factor for schizophrenia: how important is it? *Schizophr Res* 2009; **114**: 1–5.

13 Kwok W. Is there evidence that social class at birth increases risk of psychosis? A systematic review. *Int J Soc Psychiatry* 2014; **60**: 801–8.

14 Hollingshead AA. Four-factor index of social status. *Unpublished manuscript* 1975.

15 Rasic D, Hajek T, Alda M, Uher R. Risk of mental illness in offspring of parents with schizophrenia, bipolar disorder, and major depressive disorder: a meta-analysis of family high-risk studies. *Schizophr Bull* 2014; **40**: 28–38.

16 Maxwell ME. Manual for the FIGS. 1992.

17 Beards S, Gayer-Anderson C, Borges S, Dewey ME, Fisher HL, Morgan C. Life events and psychosis: a review and meta-analysis. *Schizophr Bull* 2013; **39**: 740–7.

18 Brugha TS, Cragg D. The List of Threatening Experiences: the reliability and validity of a brief life events questionnaire. *Acta Psychiatr Scand* 1990; **82**: 77–81.

19 Gurillo P, Jauhar S, Murray RM, MacCabe JH. Does tobacco use cause psychosis? Systematic review and meta-analysis. *Lancet Psychiatry* 2015; **2**: 718–25.

20 Marconi A, Di Forti M, Lewis CM, Murray RM, Vassos E. Meta-analysis of the Association Between the Level of Cannabis Use and Risk of Psychosis. *Schizophr Bull* 2016; **42**: 1262–9.

21 Varese F, Smeets F, Drukker M, *et al.* Childhood adversities increase the risk of psychosis: a meta-analysis of patient-control, prospective- and cross-sectional cohort studies. *Schizophr Bull* 2012; **38**: 661–71.

22 Bernstein DP, Stein JA, Newcomb MD, *et al.* Development and validation of a brief screening version of the Childhood Trauma Questionnaire. *Child Abuse Negl* 2003; **27**: 169–90.

23 Yan C, Cao Y, Zhang Y, Song L-L, Cheung EFC, Chan RCK. Trait and state positive emotional experience in schizophrenia: a meta-analysis. *PLoS One* 2012; **7**: e40672.

24 Gard DE, Gard MG, Kring AM, John OP. Anticipatory and consummatory components of the experience of pleasure: A scale development study. *J Res Pers* 2006; **40**: 1086–102.

25 Office For National Statistics, National Records Of Scotland, Northern Ireland Statistics And Research Agency. 2011 Census aggegate data (Data downloaded: 1 June 2016). *UK Data Service* 2016. DOI:10.5257/census/aggregate-2011-1.

26 Eurostat (European Commission). Labour Force Survey in the EU, candidate and EFTA countries — Main characteristics of national surveys, 2014. Eurostat, 2015.

27 van Nierop M, van Os J, Gunther N, *et al.* Phenotypically continuous with clinical psychosis, discontinuous in need for care: evidence for an extended psychosis phenotype. *Schizophr Bull* 2012; **38**: 231–8.

28 Department of Economic and Social Affairs of the United Nations. 2018 Revision of World Urbanization Prospects. https://population.un.org/wup/ (accessed Aug 5, 2019).

29 Vuoksimaa E, Koskenvuo M, Rose RJ, Kaprio J. Origins of handedness: a nationwide study of 30,161 adults. *Neuropsychologia* 2009; **47**: 1294–301.

30 Wicks S, Hjern A, Gunnell D, Lewis G, Dalman C. Social adversity in childhood and the risk of developing psychosis: a national cohort study. *Am J Psychiatry* 2005; **162**: 1652–7.

31 Ng M, Freeman MK, Fleming TD, *et al.* Smoking prevalence and cigarette consumption in 187 countries, 1980-2012. *JAMA* 2014; **311**: 183–92.

32 Compton WM, Han B, Jones CM, Blanco C, Hughes A. Marijuana use and use disorders in adults in the USA, 2002-14: analysis of annual cross-sectional surveys. *Lancet Psychiatry* 2016; **3**: 954–64.

33 Stevens G, Flaxman S, Brunskill E, *et al.* Global and regional hearing impairment prevalence: an analysis of 42 studies in 29 countries. *Eur J Public Health* 2013; **23**: 146–52.

34 Manera V, Antelmi L, Zeghari R, Ayache N, Lorenzi M, Robert P. Prevalence of lack of interest and anhedonia in the general population of the UK Biobank. 2019; published online July.

35 Organisation for Economic Co-operation and Development. https://stats.oecd.org/Index.aspx?DataSetCode=RPOP# (accessed Aug 7, 2019).

36 Pedersen CB, Raaschou-Nielsen O, Hertel O, Mortensen PB. Air pollution from traffic and schizophrenia risk. *Schizophr Res* 2004; **66**: 83–5.
